# Long-Chain Alkylthio Cyclodextrin Derivatives for Modulation of Quorum-Sensing-Based Bioluminescence in *Aliivibrio fischeri* Model System

**DOI:** 10.3390/ijms25137139

**Published:** 2024-06-28

**Authors:** Éva Fenyvesi, Zsófia Berkl, Laura Ligethy, Ildikó Fekete-Kertész, Márton Csizmazia, Milo Malanga, István Puskás, Levente Szőcs, Róbert Iványi, István Kese, Erzsébet Varga, Lajos Szente, Mónika Molnár

**Affiliations:** 1 CycloLab Cyclodextrin R&D Laboratory Ltd., Illatos út 7, 1097 Budapest, Hungary; milo.malanga@carbohyde.com (M.M.); puskas@cyclolab.hu (I.P.); levente.szocs@cyclolab.hu (L.S.); robert.ivanyi@cyclolab.hu (R.I.); istvan.kese@cyclolab.hu (I.K.); erzsebet.varga@cyclolab.hu (E.V.); szente@cyclolab.hu (L.S.); 2 Department of Applied Biotechnology and Food Science, Budapest University of Technology and Economics, Műegyetem rkp. 3, 1111 Budapest, Hungary; berkl.zsofia@vbk.bme.hu (Z.B.); lalaligethy@hotmail.com (L.L.); fekete.kertesz.ildiko@vbk.bme.hu (I.F.-K.); csmarton93@gmail.com (M.C.)

**Keywords:** *Aliivibrio fischeri*, alkylthio cyclodextrins, bioluminescence, quorum quenching, quorum sensing

## Abstract

Quorum sensing (QS) allows bacteria to coordinate their activities by producing and detecting low-molecular-weight signal molecules based on population density, thereby controlling the infectivity of bacteria through various virulence factors. Quorum-sensing inhibition is a promising approach to tackle bacterial communication. Cyclodextrins (CDs) are a class of cyclic oligosaccharides that reversibly encapsulate the acyl chain of the signal molecules, thereby preventing their binding to receptors and interrupting bacterial communication. This results in the inhibition of the expression of various properties, including different virulence factors. To examine the potential quorum-quenching (QQ) ability of newly prepared cyclodextrin derivatives, we conducted short-term tests using *Aliivibrio fischeri*, a heterotrophic marine bacterium capable of bioluminescence controlled by quorum sensing. α- and β-cyclodextrins monosubstituted with alkylthio moieties and further derivatized with quaternary ammonium groups were used as the test agents. The effect of these cyclodextrins on the quorum-sensing system of *A. fischeri* was investigated by adding them to an exponential growth phase of the culture and then measuring bioluminescence intensity, population growth, and cell viability. Our results demonstrate that the tested cyclodextrins have an inhibitory effect on the quorum-sensing system of *A. fischeri*. The inhibitory effect varies based on the length of the alkyl chain, with alkylthio substitution enhancing it and the presence of quaternary ammonium groups decreasing it. Our findings suggest that cyclodextrins can be a promising therapeutic agent for the treatment of bacterial infections.

## 1. Introduction

Antibiotics have been a significant discovery in the field of medical sciences. However, due to the rising rates of bacterial resistance to current antibiotics and the lack of new antibiotics, there is a growing interest in developing new treatment strategies to combat bacterial infections caused by multidrug-resistant strains [1].

The formation of biofilms by microbial species like *Pseudomonas aeruginosa* [2,3] is a significant problem that has gained interest in recent years for research on the prevention and weakening of biofilms [4,5]. Bacterial pathogens can cause disease by regulating the expression of virulence factors through a complex process [5]. They achieve this by working together through releasing, sensing, and responding to various signal molecules [6]. This process is called quorum sensing (QS), which involves the detection and response to population density by activating different types of genes. QS enables bacteria to behave as a single organism, facilitating host colonization, biofilm formation, defense against competitors, and adaptation to changing environments [7,8].

Quorum sensing was first discovered in the heterotrophic marine bioluminescent bacterium *A. fischeri* in the early 1970s [9]. Since then, extensive research has been conducted on the mechanism of bioluminescence, the genes required for controlling it, and the main QS signal, N-3-oxohexanoyl-L-homoserine lactone. As a result of these studies, *A. fischeri* has become one of the most commonly used model organisms for researching QS [10,11,12].

**Figure 1:**
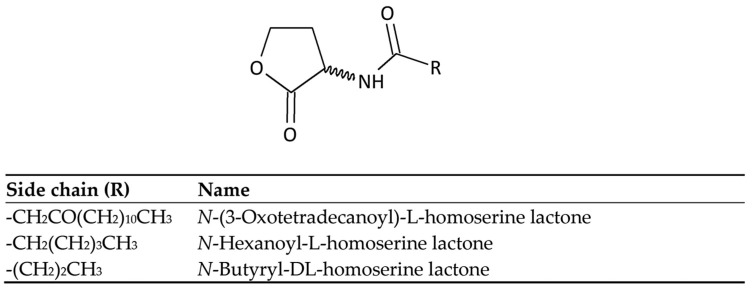
Examples of AHLs.

Gram-positive and Gram-negative bacteria use different types of signal molecules to communicate with each other through quorum sensing (QS). Gram-positive bacteria typically produce autoinducer peptides (AIP), whereas Gram-negative bacteria mainly produce N-acyl-homoserine lactone (AHL) signals. These signals vary in length and can range from 4 to 18 carbon atoms, with different substitutions on the β-position [13]. Figure 1 illustrates several examples of AHLs.

QS inhibitors either prevent the formation of these signal molecules or block their interaction with related receptor proteins, thus inhibiting QS and related gene expression. The bioluminescence inhibition bioassay was utilized to investigate the potential quorum-quenching (QQ) effects of several compounds, namely, L-prolinol, D-prolinol, furaneol acetate, sulfonylureas, sulfamethoxypyridazine, DL-pyroglutamic acid, and (Z-)-4-bromo-5-(bromomethylene)-2(5H)-furanone, on the *A. fischeri* model organism [14,15,16]. However, there is little information available in the literature regarding the impact of cyclodextrins (CDs) on QQ in *A. fischeri*. CDs are cyclic oligosaccharides composed of α(1,4)-linked D-(+)glucopyranose units. The most studied CDs are the α-, β-, and γ-CDs with six, seven, or eight glucopyranose units [17]. These molecules possess a hydrophobic inner cavity and hydrophilic outer surface. Their special property is the forming of non-covalent host–guest complexes with molecules of less hydrophilic character through the inclusion of at least some moieties or the molecule as a whole into the hydrophobic cavity based on geometrical fit [18].

Cyclodextrins have been found to effectively trap signal molecules, thereby providing a potential avenue for inhibiting QS. This area of research remains relatively unexplored in the field of pharmaceutical nanotechnology and offers significant promise for further investigation [19].

CDs were found to have a significant effect on QS pathways in *Aliivibrio fischeri*, *Serratia marcescens*, *Chromobacterium violaceum*, and *Pseudomonas aeruginosa* model organisms [20,21,22]. The effect of native CDs and their derivatives in the *A. fischeri* bioluminescence model system was first confirmed by Molnár et al. (2021) [23]. This study revealed a high QQ effect (over 60% inhibition of bioluminescence) for native α-CD at 10 mM concentration after 120 min exposure time. In addition, numerous α- and β-CD derivatives inhibited the QS-based processes, whereas the native γ-CD and its derivatives were inefficient [21,23,24,25,26,27,28,29,30,31]. The highest QS inhibition was observed by Morohoshi et al. (2013) for 6-monosubstituted alkylamino CD derivatives. It was observed that the association constants of the CD signal inclusion complexes increased with the length of the alkylamino moiety [21]. These derivatives, however, have low water solubility owing to their long alkyl side chains.

In this study, we aimed to synthesize water-soluble CD derivatives with higher ability to bind with AHLs that could be obtained through a simpler synthetic process than the preparation of the alkylamino derivatives. α- and β-CDs were monosubstituted with thioalkyl groups using good leaving groups, such as bromide for α-CD and tosyl for β-CD, according to the synthetic strategies reviewed by Kasal and Jindrich (2021) [32].

For solubility enhancement, CDs were further substituted with quaternary amino (QA) moieties at random positions. Bacterial cell walls are built up from different types of negatively charged molecules owing to the dissociation of acidic moieties such as carboxyl and phosphate. Therefore, it was expected that compounds with positive charges might penetrate the cell wall and disturb its function [33,34]. Thus, the substitution of CDs by positive QA groups can not only change their pharmacokinetic properties (solubility and bioavailability), but it may also serve as a new possible attacking weapon against bacteria.

**Figure 2:**
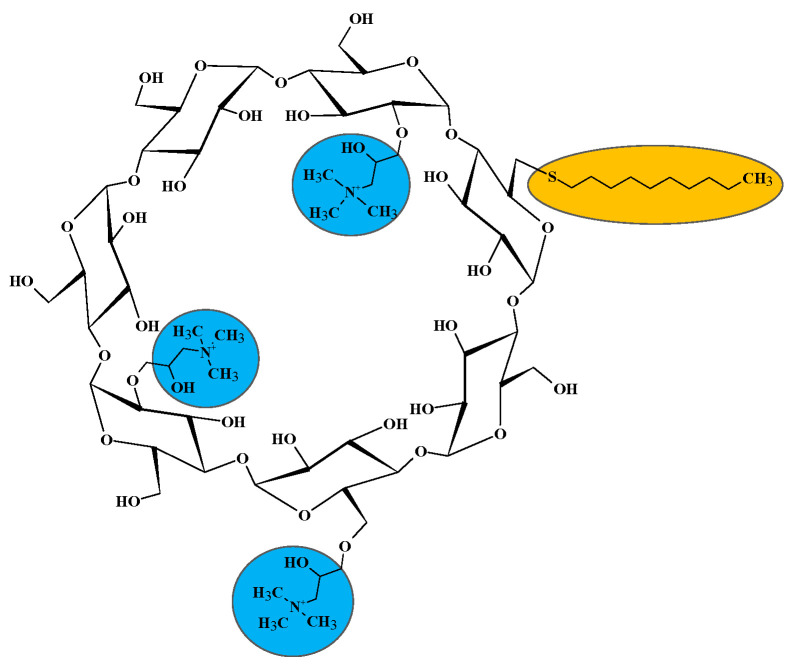
The structural formula of *N*,*N*,*N*-trimethyl-ammoniopropyl-(6-monodeoxy-6-monodecylthio)-β-CD. The 1–4 QA moieties in a molecule are located at random positions coupled to either C2, C3, or C6 OH groups, and the thioalkyl substituent is in the C6 position.

In our research work, we present the synthesis, characterization, and biological evaluation of various α- and β-CD derivatives. These derivatives contain mono-alkylthio substitution and 2−4 quaternary amino moieties in each molecule. Figure 2 demonstrates the structure of QA-(6-monodeoxy-6-monodecylthio)-β-CD as an example. Our goal was to investigate the quorum-quenching (QQ) ability of these CD derivatives as a function of concentration and time. These derivatives resemble the AHL signal molecules, each having one monoalkyl substituent. The CDs were expected not to affect bacterial viability but to disturb the control mechanisms of bioluminescence by *A. fischeri*.

The study investigated the possible cytotoxic effect of the CD derivatives to determine whether they affected bacterial viability. Using CDs substituted with both thioalkyl and quaternary amino moieties to modify the QS control mechanisms of bioluminescence in *A. fischeri* is a unique approach not found in the scientific literature, compared to native α- and β-CDs.

## 2. Results

### 2.1. Preparation of Randomly Substituted Quaternary Amino 6-Monodeoxy-6-Monoalkylthio CD Derivatives

At first, to obtain 6-monodeoxy-6-mono-decylthio-β-CD (6S-C10-β-CD), 6-monotosyl-β-CD was reacted with 1-decanethiol in a solvent (*N,N*-dimethylformamide) under an inert atmosphere using sodium methoxide.

**Figure 3:**
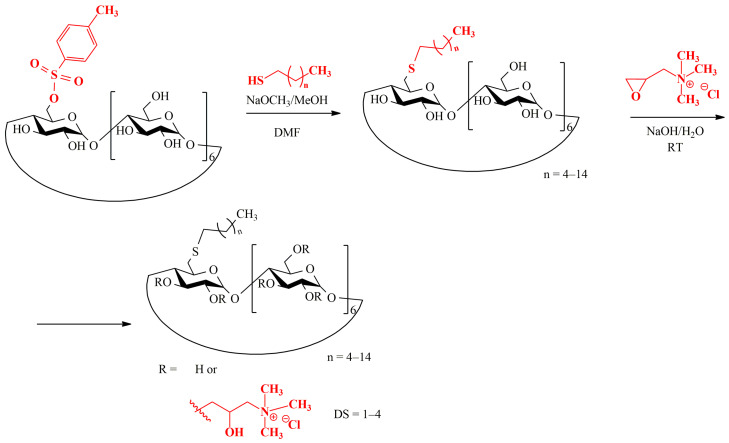
Scheme of the preparation of 6-monoalkylthio β-CD derivatives and their substitution with *N*,*N*,*N*-trimethylammoniopropyl moieties in random positions.

Similar reactions were carried out to obtain 6-monodeoxy-6-mono-dodecylthio-β-CD (6S-C12-β-CD) (**7**) and 6-monodeoxy-6-mono-hexadecylthio-β-CD (6S-C16-β-CD). The products were subjected to purification by chromatography when needed, and in the second step, they were reacted with glycidyltrimethylammonium chloride to build in positive charges (quaternary amino groups = QA) and obtain QA-6S-C10-β-CD (**8**), QA-6S-C12-β-CD and QA-6S-C16-β-CD (**9**) (Figure 3). QA-β-CD (**6**) was also used for comparison.

**Figure 4:**
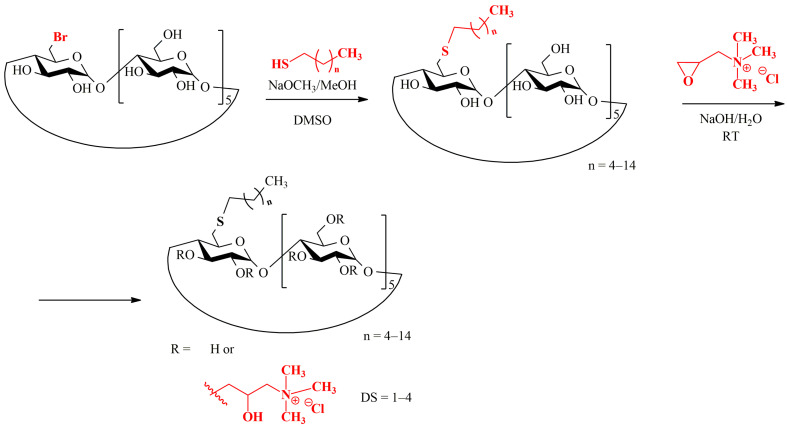
Scheme of the preparation of α-CD 6-monoalkylthio derivatives and their substitution with *N*,*N*,*N*-trimethylpropyl groups at random positions.

For the α-CD series, another reaction route was applied (Figure 4). 6-monobrominated α-CD, obtained by reacting native α-CD with N-bromosuccinimide and triphenylphosphine, was used as starting material. By strictly controlling the temperature, only one single unit of halogen can be substituted on the primary rim of α-CD. To obtain 6-monodeoxy-6-monoalkylthio α-CD derivatives, 6-monobromo-α-CDs was reacted with the proper alkanethiol in dimethylsulfoxide (DMSO) in the presence of sodium methoxide.

In order to achieve quantitative conversion, an excess of thiol was used. The reaction crudes were purified by crystallization from methanol. This reaction route was applied for the preparation of 6-monodeoxy-6-monodecylthio and 6-monodeoxy-6-monododecylthio α-CD (**3**). Permanent positive charges (quaternary amino, QA groups) were randomly built in the 6-monodeoxy-6-monoalkylthio α-CD derivatives under aqueous alkaline conditions with glycidyltrimethylammonium chloride.

The presence of positive charges on the CD units significantly enhanced the aqueous solubility of the CD derivatives, thus allowing for complete purification by dialysis. QA-(6-monodeoxy-6-monododecylthio)-α-CD (QA-6S-C12-α-CD) was obtained, and the structure elucidated by NMR and MS. QA-α-CD (**2**) was also used for comparison.

### 2.2. NMR Characrterization

**Figure 5:**
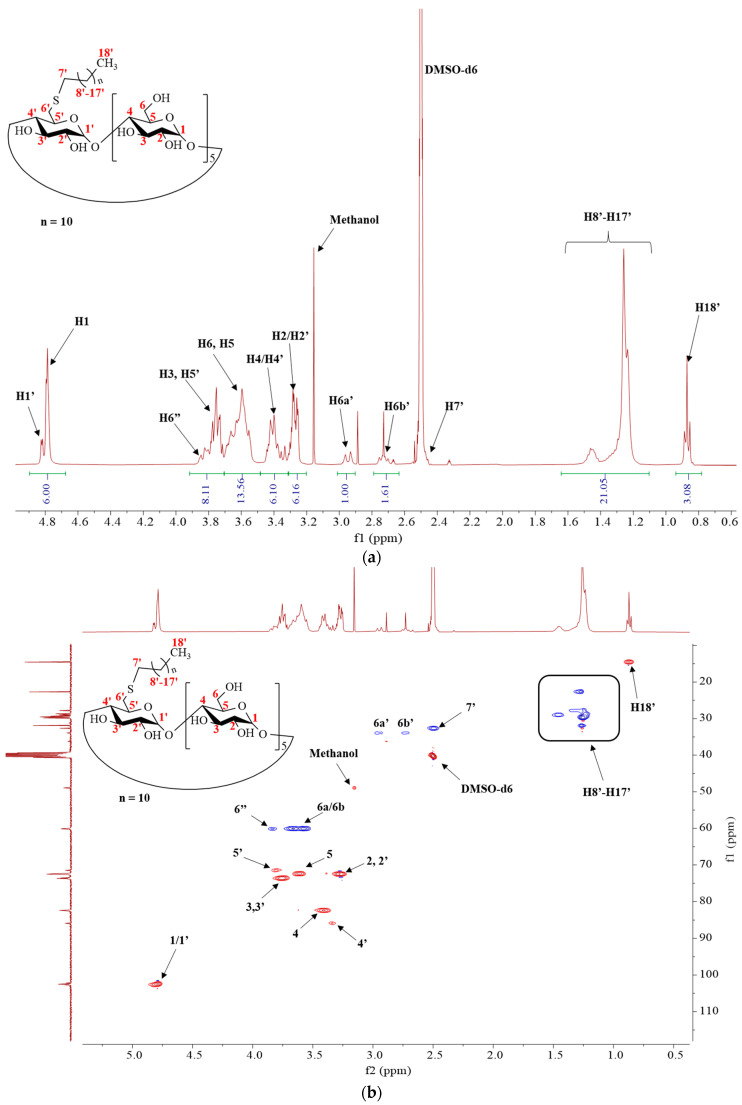
(**a**) 1H NMR spectrum of 6-monodeoxy-6-monododecylthio-α-CD (DMSO-d6, 600 MHz); δ 4.79 (6H, H1, H1′, d), 3.86–3.50 (22H, H3, H3′, H5, H6a,b, m), 3.47–3.23 (12H, H2, H2′, H4, H4′, m), 2.95 (1H, H6′b, d), 2.66 (1H, H6′a, m), 2.50 (2H, H7′), 1.46–1.24 (20H, H8′-H17′, m), 0.86 (3H, H18′,t). (**b**) DEPT-edited HSQC spectrum of 6-monodeoxy-6-monododecylthio-α-CD (DMSO-d6, 600 MHz).

**Figure 6:**
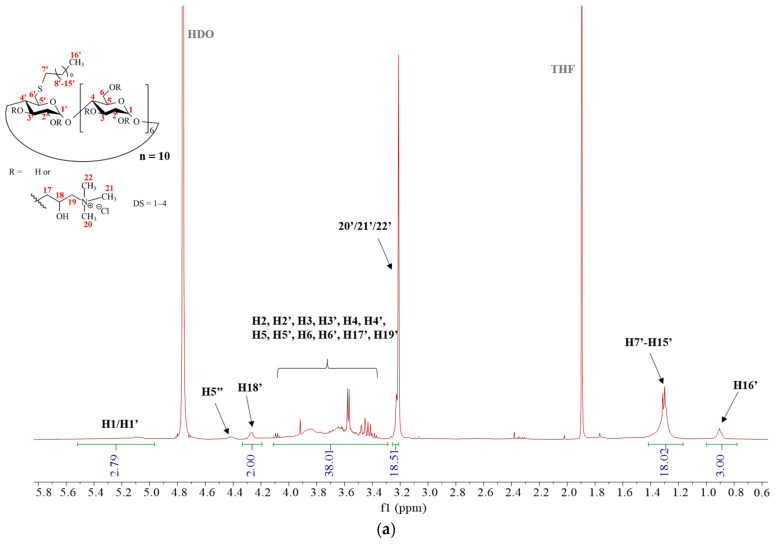

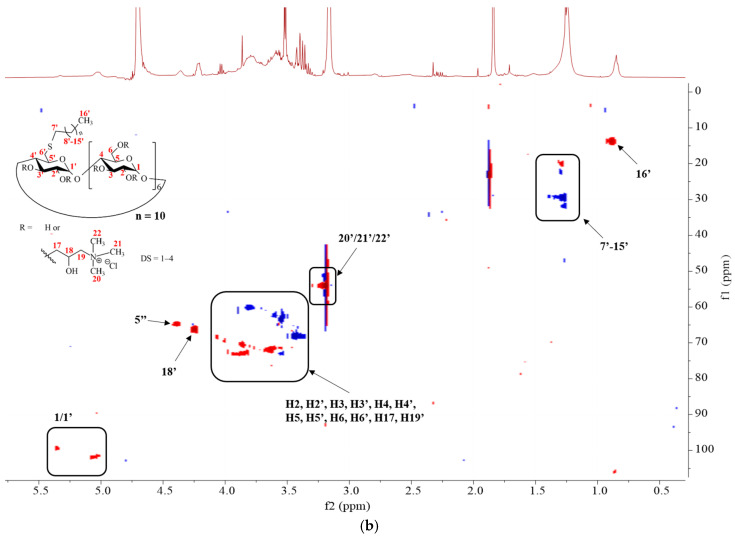
(**a**) ^1^H NMR spectrum of QA-6S-C10-β-CD (**8**) (D_2_O, 600 MHz); δ (ppm): 5.50-4.95 (7H, H1, H1′, br d), 4.42 (1H, H5″, s), 4.27 (1H, H18, m), 4.10-3.30 (38H, H2, H2′, H3, H3′, H4, H4′, H5, H5′, H6, H6′, H17, H19, m), 3.21 (18H, H20, H21, H22, d), 1.30 (18H, H7′-H15′, m), 0.91 (3H, H16′, t). (**b**) DEPT-edited HSQC spectrum of QA-6S-C10-β-CD (**8**) (D_2_O, 600 MHz).

The NMR spectra of 6S-C12-α-CD (**3**) and QA-6S-C10-β-CD (**8**) can be seen in Figure 5a,b and Figure 6a,b. In the 1H NMR spectrum of 6S-C12-α-CD (**3**) (Figure 5a,b), the assignations belong to the following protons (both ^1^H and HSQC spectra were recorded in DMSO-d6):

- The signals of the anomeric protons can be identified at around 4.8 ppm;
- The core region of the CD ring lays in the range of 3.9 and 3.2 ppm;
- The proton signals of the aliphatic side chain are assigned in the range of 1.6 and 0.8 ppm;
- The methyl group of the aliphatic side chain is located at 0.86 ppm;
- At 2.50 ppm, the signal of DMSO (residual solvent) can be recognized.

The full assignment of 6S-C12-α-CD (**3**) is shown in Figure 5a,b.

After quaternarization, the spectrum becomes more chaotic, since a mixture of isomers is formed. The calculated degree of substitution (DS) is two for quaternary ammonium groups.

For internal reference of the DS calculation, the integrals of the anomeric protons could not be used; thus, H16′, the methyl moiety terminating the alkyl chain, was selected for this purpose. In addition to the regions mentioned above, new peaks can be seen in the spectra (both ^1^H and HSQC spectra were recorded in D_2_O):

- The methyl groups of the quaternary amino moieties can be recognized at around 3.3 ppm with high intensity;
- The signals of methine in the quaternary amino groups are located at around 4.3 ppm;
- The signals at around 4.76 and 1.9 ppm belong to the two residual solvents: HDO (used as internal standard) and tetrahydrofurane (THF), respectively.

The full assignment of the spectra for QA-6S-C10-β-CD (**8**) can be seen in Figure 6a,b.

**Figure 7:**
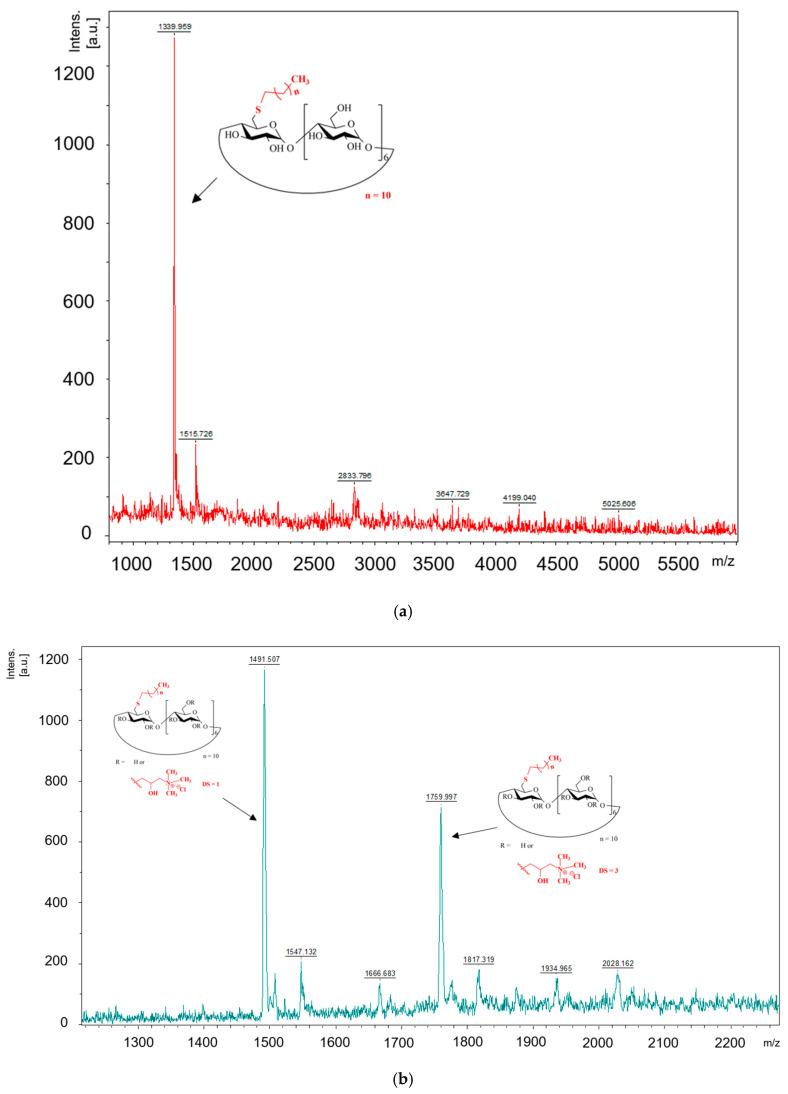
(**a**) MALDI spectra of 6S-C12-β-CD (**7**). (**b**) MALDI spectrum of QA-6S-C10-β-CD (**8**).

Figure 7a,b show the MALDI spectra of 6S-C12-β-CD (**7**) and QA-6S-C10-β-CD (**8**).

The main peaks can be assigned to the molecule ions of 6S-C16-β-CD substituted with one and three QA moieties (QA-6S-C16-β-CD (**9**)) following the results of the NMR study, which gave DS 2 as an average.

### 2.3. Characterization of Aggregation

**Figure 8:**
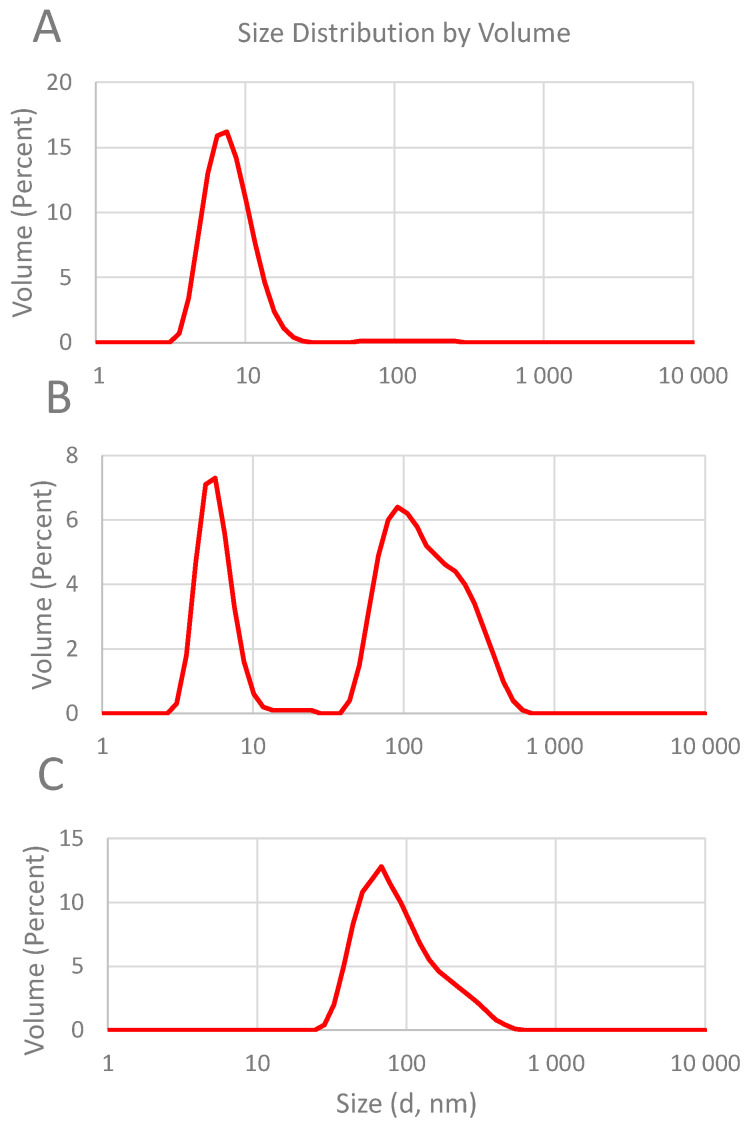
Size distribution curves recorded in 1% aqueous solutions of the following CD derivatives (average of 5 parallel measurements). (**A**): QA-6S-C10-α-CD (**4**); (**B**): QA-6S-C10-β-CD (**8**); (**C**): QA-6S-C16-β-CD (**9**).

Most CDs are prone to aggregation in aqueous solution due to their numerous hydroxyl moieties [35]. For characterization of the aggregation behavior, the volume particle size distribution curves were recorded for an α-CD derivative and two β-CD derivatives (Figure 8). The non-aggregated CDs have a size (i.e., hydrodynamic-equivalent spherical diameter) of approx. 1.5 nm, corresponding to the physical dimensions of a hydrated single cyclodextrin molecule. In the aqueous solutions of the QA-alkylthio-CD derivatives, non-aggregated species cannot be detected due to the introduction of the apolar alkyl moiety; only aggregated species can be identified. As the size of the alkyl chains increases, these alkylthio derivatives tend to aggregate to a higher extent. Relatively small-sized aggregates of 8-10 nm (average 8.2 nm) characterized with monomodal size distribution were formed by the QA-6S-C10-α-CD (**4**) compound. On the contrary, the size distribution curve for QA-6S-C10-β-CD (**8**) was bimodal: in addition to a relatively uniform population of small particles of 8–10 nm average diameter, larger aggregates with high polydispersity of approx. 50–500 nm size (average 163 nm) can also be observed. The QA-6S-C16-β-CD (**9**) sample demonstrated even more pronounced self-association: neither non-aggregated nor small-sized fractions can be observed; only large-sized particles of 50–500 nm (average 111 nm) of high polydispersity were detected.

**Figure 9:**
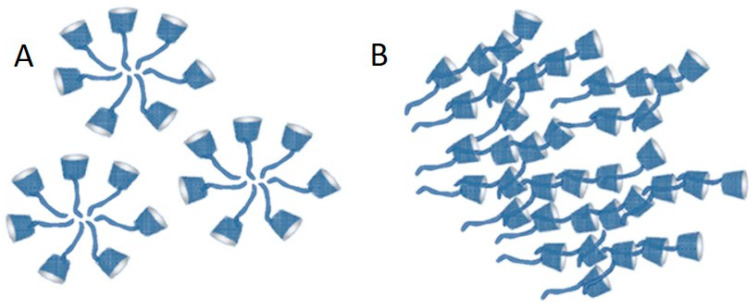
Scheme of small-sized micelle-like aggregates (**A**) and bigger-sized aggregates (**B**) of 6-monodeoxy-6-monoalkylthio CD derivatives formed by self-assembly via supramolecular interactions.

Similar size distribution curves were observed for the other derivatives, too. It has been observed that derivatives containing longer alkyl side chains may not produce clear solutions due to the formation of large-size aggregates. Figure 9 shows the schemes of two main types of aggregates.

### 2.4. Bioluminescence Inhibition Assay in the Aliivibrio fischeri Model System

#### 2.4.1. Effect of α-Cyclodextrins on Bioluminescence

**Figure 10:**
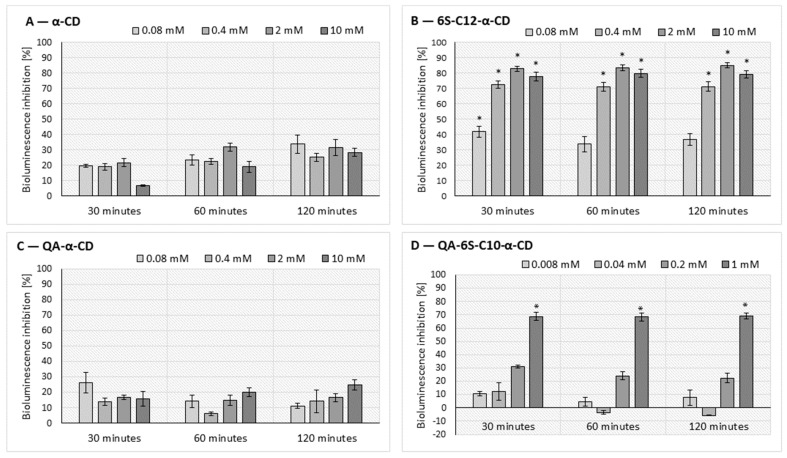
Effect of α-CD (**A**), 6S-C12-α-CD (**B**), QA-α-CD (**C**), and QA-6S-C10-α-CD (**D**) on the bioluminescence intensity of the *A. fischeri*. Significant inhibition after 30, 60, and 120 min of exposure is marked by an asterisk (*) (*p* < 0.05), as determined by the Tukey HSD test. Data represent averages of four replicates.

The quorum-quenching (QQ) effect of α-CD (**1**), QA-α-CD (**2**), and their alkylthio derivatives, 6S-C12-α-CD (**3**) and QA-6S-C10-α-CD (**4**), was determined through the measurement of bioluminescence intensity, population growth, and viability of the *A. fischeri* Gram-negative bacteria. The α-CD (**1**), QA-α-CD (**2**), 6S-C12-α-CD (**3**), and QA-6S-C10-α-CD (**4**) affected bioluminescence in the tested concentrations, as presented in Figure 10.

The CD concentration, exposure time, and substitution significantly influenced the QQ effect of the tested α-CDs. As illustrated in Figure 10, the 2 mM 6S-C12-α-CD (**3**) had the highest inhibition (85%, 120 min), but this CD also proved an effective QQ agent after 30 and 60 min of exposure (in the 0.4–10 mM concentration range).

We measured significant inhibition (68%) for 1 mM QA-6S-C10-α-CD (**4**) at each time point, but no significant inhibition was observed in the case of the α-CD (**1**) and QA-α-CD (**2**) in the examined period. For the alkylthio derivatives, increasing inhibition was measured proportionally with time, suggesting the contribution of other influencing parameters in the test system and the decreased availability of the signal(s).

The results of repeated measures variance analysis (RM ANOVA) of the α-CD variants also demonstrated the significant QQ efficiency of these CDs (Table 1).

In the case of the alkylthio derivatives, both the contact time and the CD treatment affected bacterial communication, and thus the bioluminescence intensity of the model organism. For the α-CD and the QA-α-CD, the exposure time and the combined effect of the exposure time and the CD treatment also significantly affected bioluminescence.

#### 2.4.2. Effect of α-Cyclodextrins on Cell Viability

Cell growth in the population (optical density) was determined after 30, 60, and 120 min of incubation (Supplementary Figure S1). The experiment showed that α-CD (**1**), QA-α-CD (**2**), and QA-6S-C10-α-CD (**4**) inhibited population growth by up to 30% within 30, 60, and 120 min, regardless of the concentration. However, 6S-C12-α-CD (**3**) caused inhibition increased in proportion to the concentration, at 10 mM up to 50%, 54%, and 59% inhibition after 30, 60, and 120 min of exposure, respectively. This finding highlights the potential detrimental effect of CDs on cell viability, which could affect the QS process. Therefore, the potential cytotoxic effect of CDs was also determined using the tetrazolium reduction-based cell viability assay.

The cell viability assay characterizing the activity of the hydrogen transport (the dehydrogenase enzyme activity) is based on the spectrophotometric measurement of the absorbance of a formazan product of the reduction in the tetrazolium salts.

The absorbance—which we determined after 120 min of exposure—is directly proportional to the metabolic activity of the cells. According to the results (Supplementary Figure S2), 10 mM of α-CD (**1**) and QA-α-CD (**2**) induced 29 and 41% inhibition in the viability of *A. fischeri*. However, in the case of 6S-C12-α-CD (**3**), we measured a maximum 40% (at 2 and 10 mM) decrease in cell viability, increasing proportionally with the concentration; QA-6S-C10-α-CD (**4**) caused 24–27% inhibition of the enzyme activity, regardless of concentration.

The results of the cell viability examinations (population growth and hydrogen transport activity) indicated that the QQ effect of the novel alkylthio cyclodextrin derivatives is partly due to their cytotoxic nature.

This suggests further studies to assess the extent and nature of the cytotoxic effect, including the examination of the activity of other essential enzymes, the production of reactive oxygen species, the integrity of the cell membrane, and the number of viable cells.

#### 2.4.3. Effect of β-Cyclodextrins on the Bioluminescence

**Figure 11:**
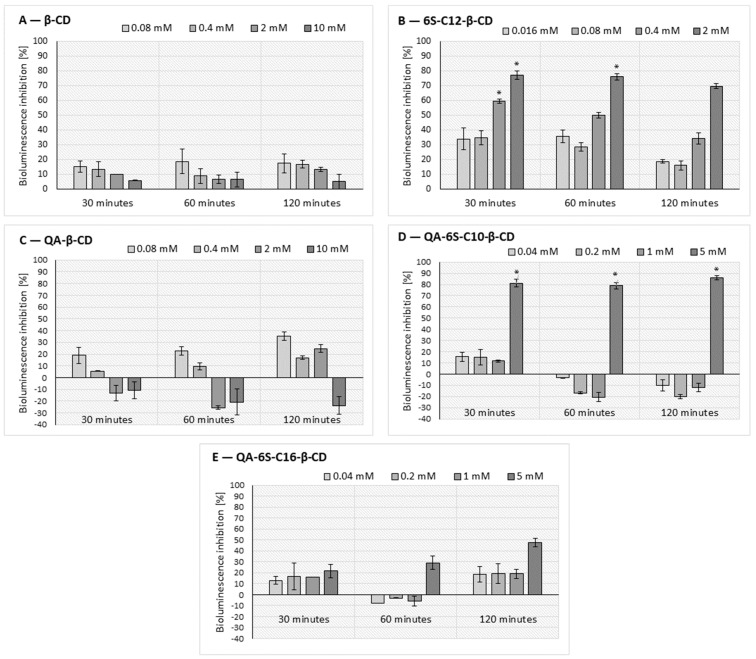
Effect of β-CD (**A**), 6S-C12-β-CD (**B**), QA-β-CD (**C**), QA-6S-C10-β-CD (**D**), and QA-6S-C16-β-CD (**E**) on the bioluminescence intensity of the *A. fischeri*. Significant inhibition after 30, 60, and 120 min of exposure is marked by an asterisk (*) (*p* < 0.05), as determined by the Tukey HSD test. Data represent averages of four replicates.

Similarly to the α-CDs, the potential QQ and cytotoxic effect of β-CD (**5**), QA-β-CD (**6**), 6S-C12-β-CD (**7**), QA-6S-C10-β-CD (**8**), and QA-6S-C16-β-CD (**9**) were determined through the measurement of bioluminescence intensity, population growth, and viability of the *A. fischeri* model bacteria. The increasing concentrations of the β-cyclodextrin variants affected the bioluminescence, as presented in Figure 11. It could be stated that the QQ effect of the β-CDs was significantly affected by the cyclodextrin concentration, exposure time, and alkylthio substitution. However, the degree of inhibition was generally lower than that of the α-CDs.

As shown in Figure 11, 5 mM QA-6S-C10-β-CD (**8**) exhibited the highest significant QQ effect (86%, 120 min). However, at lower concentrations (0.04–1 mM), this alkylthio derivative had a slight, statistically not significant (maximum 21%) stimulatory effect on the bioluminescence after 30 and 60 min of contact time. The 6S-C12-β-CD (**7**) derivative also exerted an outstanding inhibition proportionally with the concentration at all time points, with a maximum of 77% (2 mM, 30 min). However, the inhibitory effect did not exceed 35% for β-CD (**5**) and QA-β-CD (**6**) in any case, and these slight effects were not statistically significant.

As illustrated, in the case of the β-CDs, the longest examined contact time (120 min) and the highest examined CD concentrations (0.4–5 mM) resulted in significant bioluminescence inhibition. It was clearly demonstrated that—similarly to the α-CDs—the alkyl substitution increased the QQ effect in the case of the β-CDs, but this improvement did not increase proportionally to the length of the alkyl chain. This is strongly supported by the comparative assessment of the QQ potential of the QA-6S-C10-β-CD (**8**) and QA-6S-C16-β-CD (**9**) derivatives. The results of the RM ANOVA analysis of the β-CD variants (Table 2) suggested that the 6S-C12-β-CD (**7**), QA-β-CD (**6**), and QA-6S-C10-β-CD (**8**) treatment significantly influenced the bacterial communication, and thus the bioluminescence emission of the bacteria.

As shown in Table 2, the exposure time proved to be a significant influencing factor of the QQ efficiency of most of the studied β-CDs (except for 6S-C12-β-CD (**7**)).

The combined effect of the exposure time and the CD treatment proved significant only in the case of 6S-C12-β-CD (**7**) and QA-6S-C16-β-CD (**9**). Unlike the α-CD variants, there were no cases in which all three factors—the exposure time, the CD treatment, and their combination—proved to cause significant differences in the bacterial communication compared to the control.

#### 2.4.4. Effect of β-Cyclodextrins on Cell Viability

The β-CD (**5**) and QA-β-CD (**6**) exerted inhibition on population growth (Supplementary Figure S3). However, their degree of inhibition proportionally decreased with concentration at each time point, with a maximum of 34% (at 30 min of exposure, 0.08 mM). Similar trends were observed for QA-6S-C10-β-CD (**8**) and QA-6S-C16-β-CD (**9**) after 30 and 60 min of contact, but after 120 min, the highest tested concentrations (5 mM) showed a greater inhibitory effect (47 and 38%, respectively). The 6S-C12-β-CD (**7**) derivative showed a high inhibition (maximum 72, 69, and 65% at 2 mM, after 30, 60, and 120 min) of cell proliferation, proportionally increasing with the concentration. Based on the results of the tetrazolium reduction-based cell viability assay (Supplementary Figure S4), β-CD (**5**) and QA-β-CD (**6**) had a minor (maximum 15 and 10%, 120 min) inhibitive effect on cell viability at the lowest concentrations (at 0.08 mM). The highest inhibition (64%, 120 min) was achieved by 5 mM QA-6S-C10-β-CDP (**8**). However, a vitality-reducing effect (35 and 41%) was observed for 5 mM QA-6S-C16-β-CD (**9**) and 2 mM 6S-C12-β-CD (**7**) as well. Similarly to the α-CDs, these results suggest that the β-CD variants may have a cytotoxic effect, potentially interfering with the QQ effect and requiring further investigation.

## 3. Discussion

### Comparative Evaluation of Cyclodextrin Effectiveness

Previous studies have shown the quorum-quenching effects of various cyclodextrins in quorum-sensing bacterial model systems other than *A. fischeri* [20,26,27,30]. However, the impact of cyclodextrins on *A. fischeri* has been marginally explored.

In our research, we designed and synthesized a range of cyclodextrin derivatives to enhance the efficiency of native CDs in inhibiting quorum sensing (QS). We exposed the derivatives for 30, 60, and 120 min. We found that the addition of 6-mono-dodecanthiol-substituted α-CD (6S-C12-α-CD (**3**)) at concentrations ranging from 0.4 to 10 mM significantly reduced the AHL-mediated bioluminescence of *A. fischeri*.

We observed a significant decrease in bioluminescence intensity after 120 min of contact time. At concentrations of 0.4, 2, and 10 mM, the intensity decreased by 71%, 85%, and 79%, respectively.

After 30, 60, and 120 min of exposure, the 2 mM concentration of 6-mono-dodecanethiol-substituted β-CD (6S-C12-β-CD (**7**)) showed significant inhibition of bacterial communication, resulting in reductions of 77%, 76%, and 70%, respectively. Additionally, both the *N*,*N*,*N*-trimethylammoniopropyl-6-mono-decanethio-substituted α-CD and β-CD derivatives (1 mM QA-6S-C10-α-CD (**4**) and 5 mM QA-6S-C10-β-CD (**8**)) significantly reduced bioluminescence after 120 min of exposure, with reductions of 69% and 86%, respectively.

Among the tested QS inhibitors, 6S-C12-α-CD (**3**) and 6S-C12-β-CD (**7**) were the most effective, as indicated by the effective concentration values (EC_20_ and EC_50_) in Table 3.

We observed that adding both *N*,*N*,*N*-trimethyl-ammoniopropyl and mono-decanethiol groups to α-CD and β-CD parent molecules enhanced their ability to inhibit quorum sensing (QS). This effect was particularly noticeable at higher concentrations (1 and 5 mM, respectively). On the other hand, adding a longer alkyl chain to QA-C16-β-CD did not result in any further improvement in QS inhibition. In fact, at 1 mM concentration, QA-C16-β-CD showed a reduced inhibitory effect of 48%.

In our previous research [23], the concentration- and time-dependent inhibition in QS-regulated bioluminescence of nine cyclodextrins (CDs) was evaluated applying an *A. fischeri* bacterial model system. Numerous derivatives including 2-hydroxypropyl (HP-α-CD, HP-β-CD), random-methylated (RAMEA, RAMEB, RAMEG), trimethyl-ammoniopropyl (QA-α-CD), and sulfobutyl-ether (SBE-β-CD), as well as their epichlorohydrin-crosslinked polymers (α-CDPS and β-CDPS), were examined alongside native α-, β-, and γ-cyclodextrins (α-CD, β-CD, and γ-CD). The results of the study revealed that the quorum-quenching (QQ) efficacy of the tested CDs was influenced by factors such as interior cavity size, structure, concentration, and duration of exposure. Notably, only α-CD, β-CD, and their derivatives were able to efficiently reduce bacterial bioluminescence. On the other hand, γ-CD was found to be ineffective, possibly due to the alkyl chain of *A. fischeri* signal molecules (N-(3-oxohexanoyl)-L-homoserine lactone (3-oxo-C6-HSL) and N-octanoyl-homoserine lactone (C8-HSL) fitting better into the narrower and smaller cavities of α-CD and β-CD compared to the higher cavity of γ-CD. Furthermore, it was observed that 2-hydroxypropyl and trimethyl-ammoniopropyl substitutions or random methylation of α-CD derivatives did not improve the QQ ability of native α-CD. However, our recent research demonstrated that a significant improvement in QQ ability was achieved through the 6-mono-dodecanethiol substitution of α-CD (6S-C12-α-CD (**3**)).

In our previous study, it was found that random methylation and 2-hydroxypropyl substitution of β-CD did not improve the QQ ability of β-CD [23]. However, SBE-β-CD showed slightly better QQ effects than native β-CD. On the other hand, when *N*,*N*,*N*-trimethyl-ammoniopropyl was randomly introduced to β-CD (QA-β-CD (**6**)), or when 6-mono-decanethiol groups were added to β-CD (QA-6S-C10-β-CD (**8**)), there was a significant increase in bioluminescence inhibition.

The QS-interfering effects of alkylamine-modified cyclodextrins have also been investigated by Morohoshi and colleagues [21] in Gram-negative bacterial systems. Regarding the prodigiosin production in *Serratia marcescens*, they found improved QS inhibitory activity compared to native α-CD, β-CD, and γ-CD molecules with 6-alkylamino-α- or γ-CDs and 2-alkylamino-CDs. Additionally, 6,6′-dioctylamino-β-CD, containing two octylamino groups, exhibited higher QQ activity than 6-mono-octylamino-β-CD.

Morohoshi et al. [21] quantitatively analyzed the interactions between the signal (N-hexanoyl- L-homoserine-lactone, C6-HSL) and CD derivatives. Synthetic β-CD derivatives with 6-alkylamino substitution exhibit higher equilibrium binding constants for homoserine lactone signal molecules than native β-CD. In addition, the synthesized CD derivatives also demonstrated strong QS inhibitory effects in other Gram-negative bacterial QS systems such as *C. violaceum* and *P. aeruginosa* [21]. However, unlike our studies, the effects of the four β-cyclodextrin derivatives on the production of prodigiosin by *S. marcescens*, violacein by *C. violaceum,* and elastase activity of *P. aeruginosa* were assessed without considering the concentration and time dependence. All the tested CD derivatives were applied at a concentration of 10 mg/mL with an extended exposure duration (18–20 h). This was different from our study, which had a maximum exposure time of 120 min. It is important to mention that various contact times are recommended for testing different bacterial systems depending on the endpoint. Additionally, we observed a notable difference in methodology compared to other studies, such as the work of Morohoshi et al. (2013) [21], who cultured inoculated cells together with β-CD derivatives for 18–20 h. In our investigation, we used a 16 h old (overnight) cell culture for testing. These differences in methodology may lead to varying results when compared to the existing literature.

New β-cyclodextrin derivatives with antimicrobial properties have been reported recently. These derivatives are effective against *Staphylococcus aureus*, *Escherichia coli*, and *Pseudomonas aeruginosa* and are inspired by the antimicrobial peptide polymyxin. Like our CD derivatives, they feature both cationic moieties (seven aminomethyltriazolyl groups) and single hydrophobic tails of varying lengths (C8–C22). However, it is still unclear how the structure of the tested β-CD derivatives contributes to their high bacteriotoxicity.

In summary, a promising strategy for quenching QS is the attachment of long alkyl chains to cyclodextrins. This mimicking function is important for the regulation of QS, as has been demonstrated in previous research. Some fatty acids have been found to mimic signal molecules of various microbes, which inhibit biofilm formation and virulence [36,37]. A similar mimicking function may also be present in monoalkyl-substituted CDs. Combining this effect with the trapping of signals within the CD cavity could lead to an effective attenuation of QS-regulated processes in various bacterial systems.

## 4. Materials and Methods

The following cyclodextrin derivatives are the products of CycloLab (Budapest, Hungary): 6-monodeoxy-6-monobromo-α-CD, 6-mono-O-tosyl-β-CD, trimethylammoniopropyl-α-CD, and β-CD. The reagents and solvents of synthesis grade were purchased from Sigma-Aldrich, St. Louis, MO, USA. A magnetic stirrer (Büchi, Flawil, Switzerland) and rotavapor (Büchi) were used in the synthesis.

Preparative chromatographic separations were performed on a Büchi preparative chromatography system using a SiliCycle SiliaCartridger (SiliCycle Inc., Quebec City, QC, Canada) 40 mm cartridge packed with LiChroprep RP-18 Phase (40–63 µm; Sigma Aldrich, St. Louis, MO, USA) reversed-phase silica as a stationary phase, a water/methanol gradient elution, and a Büchi UV Photometer C-635 as a detector.

### 4.1. Synthesis of Cyclodextrins

The 6-monodeoxy-6-monobromo-α-CD was dissolved in DMF in a 3-neck round-bottomed flask under an inert atmosphere. Then, the proper amount of alkylthiol and sodium methylate were added in sequence and stirred while being heated to 80 °C.

The reaction was monitored by thin-layer chromatography (TLC, eluent: dioxane:25% ammonia 10:7). According to the TLC, the starting 6-monodeoxy-6-monobromo-α-CD was entirely (>99%) used up, so the reaction was completed within 2 h. The solvent was removed under reduced pressure at rotavapor, and the yellowish product was precipitated with methanol to remove the unreacted reagents that remained in the mother liquor.

The solid was filtered, extensively washed with methanol to eliminate the traces of methylate and alkylthiol, then washed with acetone until a white solid was obtained, and finally dried, and constant weight in a vacuum-drying box was achieved in the presence of KOH and P_2_O_5_ as drying agents.

In the case of 6S-C10-α-CD, further purification by chromatography was necessary (isocratic elution with acetonitrile:H_2_O:25% ammonia 10:5:2).

The 6-mono-O-tosyl-β-CD and DMF were weighed in a 3-neck round-bottomed flask under an inert atmosphere, and the proper amount of alkylthiol and sodium methylate were added in sequence while stirring; the reaction mixture was then heated to 60 °C. The reaction was monitored by thin-layer chromatography (TLC, eluent: dioxane:25% ammonia:n-propanol 10:7:3) and found to be completed (>99% of 6-mono-O-tosyl-β-CD was reacted) within 2–4 h, depending on the alkylthiol (the longer the alkyl chain, the longer reaction time necessary). DMF was removed under reduced pressure at rotavapor, and the yellow product was precipitated with methanol to remove the residual alkylthiol and methylate; the solid was filtered, extensively washed with methanol until a white solid was obtained, and finally dried until constant weight in a vacuum-drying box in the presence of KOH and P_2_O_5_ as drying agents. In the case of 6S-C16-β-CD, further purification by chromatography was necessary (isocratic elution with acetonitrile:H_2_O:25% ammonia 10:5:2). The yield varied between 50% and 90%.

A part of each of the 6-monodeoxy-6-monoalkylthio CD derivatives was solubilized in aqueous alkaline solution (NaOH). The reaction mixture was cooled down (T ≈ 10–15 °C), and glycidyltrimethylammonium chloride was added in one portion and stirred overnight. The reaction was monitored by TLC (eluent: methanol:acetic acid:0.1 N ammonium acetate 10:1:9) until the disappearance of the spot of the starting material (>99% was used).

The reaction mixture was neutralized, and the volatiles were removed under reduced pressure at rotavapor. The obtained solids were washed with methanol and acetonitrile, and then extensively dialyzed for 36 h to get rid of all small molecular impurities. The dialysate was finally freeze-dried. The yield varied between 60% and 70%.

The characteristics: abbreviations (A), the molecular weight (MW), the solubility in water at 25 °C (WS), and the degree of substitution (DS) of the tested CDs are presented in Table 4 and Table 5.

### 4.2. NMR Spectra

^1^H and HSQC NMR spectra were recorded on Varian VXR-600 (Agilent Technologies, Palo Alto, CA, USA) at 298 K, equipped with a 5 mm inverse-detection gradient (IDPFG) probe head. Standard pulse sequences and processing routines available in VnmrJ 3.2C/Chempack 5.1 were used, using residual solvent signals (HDO at 4.79 ppm) as an internal reference. Thirty-two transients were coadded. Before integration, automatic baseline correction was performed.

### 4.3. MALDI-TOF Mass Spectra

The spectra were recorded on a Microflex LRF system (Bruker, Karlsruhe, Germany). The Microflex LRF operated in positive ion mode using the linear detector.

Ion generation was achieved using a 60 Hz N2-Cartridge-Laser including variable power attenuator and UV optics. The laser operated was at 337 nm, and 2,5-dihydroxybenzoic acid (DHB) was used as a matrix. For sample preparation, 2,5-dihydroxybenzoic acid was used as a matrix.

### 4.4. Aggregation Studies

The aggregation behavior (nanoparticle formation) was studied by photon correlation spectroscopy (dynamic light scattering) using Malvern Zetasizer Nano ZS (Malvern Instruments, Malvern, UK) equipment at 25 °C using 1% aqueous solution. The size distribution curves according to the volume of particles were recorded, and the average of 5 parallel measurements was calculated.

### 4.5. Examination of the Effect on Bacterial Communication

The time- and concentration-dependent effect of native α- and β-CDs, their *N*,*N*,*N*-trimethyl-ammoniopropyl derivatives, and novel alkylthio derivatives were tested in a series of short-term (30–120 min) experiments at 0.008–10 mM concentration range. To differentiate between the quorum-quenching effect and the cytotoxic effect of the CDs, bioluminescence as well as cell viability (the population growth and the enzyme activity) were determined.

#### 4.5.1. Bacterial Strains and Culture Conditions

The bacterial strain *A. fischeri* (NRRL B-111 77) was cultured and maintained in the laboratory under axenic circumstances. The 16 h old (overnight) cell culture applied for the tests was prepared by inoculating 30 mL of Photobacterium medium with an ampoule of lyophilized bacteria. The culture was shaken in the dark at 160 rpm, 24 °C, to an optical density of 0.6 at 600 nm (OD600).

The ingredients of the Photobacterium growth medium were (pH = 7.2) 30 g NaCl, 6.1 g NaH_2_PO_4_∙H_2_O, 2.75 g K_2_HPO_4_, 0.204 g MgSO_4_∙7 H_2_O, 0.5 g (NH_4_)_2_HPO_4_, 5 g peptone, 0.5 g yeast extract, and 3 cm^3^ glycerol per 1 L distilled water, as described by Fekete-Kertész et al. (2017) [38]. The measurements were performed following the description of Leitgib et al. (2007) [39], by the modification of the ISO 11348-3 protocol [40].

#### 4.5.2. Preparation of CD Stock Solutions

The tested CD molecules were suspended in sterile distilled water and sterilized in an autoclave. A dilution series was prepared from the stock solutions (suspensions), covering a 0.04–50 mM concentration range.

#### 4.5.3. Bioluminescence Inhibition Assay

The experiments were carried out with overnight *A. fischeri* cell culture in 96-well microtiter plates with a total volume of 250 µL. Fifty microliters of the members of the fivefold dilution series made from stock solutions were added to the wells of a sterile, transparent, 96-well, round-bottomed Sarstedt microtiter plate in four replicates. The concentrations of the members of the dilution series are presented in Table 6. Distilled water was used as a negative control in the same volume.

To ensure the validity of data and verify the sensitivity of the bacteria, a five-member CuSO_4_ dilution series (1.6 ppm, 8 ppm, 40 ppm, 200 ppm, and 400 ppm) was used as a positive control. After that, 200 μL of the diluted overnight culture was added to the wells, except for the blank samples, for which 200 μL of Photobacterium medium was added instead.

The bioluminescence intensity was measured with a Fluostar Optima microplate reader (BMG Labtech, Ortenberg, Germany; fluorescence detection limit > 30 amol/well ATP) after 30, 60, and 120 min of exposure. The evaluation of the results was carried out as described by Ujaczki et al. (2015) [41].

**Table 6:** The concentration of the members of the dilution series.

Cyclodextrin	Concentration [mM]			
6S-C12-β-CD (**7**)	0.016	0.08	0.4	2
QA-6S-C10-α-CD (**4**)	0.008	0.04	0.02	1
QA-6S-C10-β-CD (**8**), QA-6S-C16-β-CD (**9**)	0.04	0.2	1	5
α-CD (**1**), QA-α-CD (**2**), 6S-C12-α-CD (**3**), β-CD (**5**), QA-β-CD (**6**)	0.08	0.4	2	10

### 4.6. Optical Density Measurement—The Population Growth Assay

To investigate whether the CDs had a cytotoxic effect, the growth of the bacterial population was determined through the measurement of the optical density (OD). The measurements were carried out as described by Molnár et al. (2021) [23]. The OD was measured after the incubation period, following the bioluminescence inhibition assay with DIALAB ELx800 ELISA Microplate Reader (Dialab GmbH, Wiener Neudorf, Austria) at the wavelength of 630 nm.

### 4.7. Cell Viability—Tetrazolium Reduction Assay

To examine the potential influence of the CDs on the vitality of the bacteria, an enzyme activity assay was applied using 3-(4,5-dimethylthiazol-2-yl)-2,5-diphenyltetrazolium bromide (MTT).

The measurements were carried out as described by Molnár et al. (2021) [23], with a few modifications. Metabolically active cells reduce the green-colored MTT into a black formazan product, whose concentration is directly proportional to the activity of the viable cells. Following the population growth assay, the assay was performed in the same microplates by adding 30 μL of 4 mg/mL sterile MTT solution to each well.

The microtiter plate was then incubated for 30 min until color development. The absorbance was measured spectrophotometrically with a DIALAB ELx800 ELISA Microplate Reader (Dialab GmbH, Austria) at the wavelength of 630 nm and with a Fluostar Optima BMG Labtech microplate reader at the wavelength of 544 nm.

### 4.8. Statistical Analysis

Repeated measures analysis of variance (RM ANOVA) was performed with TIBCO Statistica™ 13.5 (TIBCO Software, Inc., Palo Alto, CA, USA) software to investigate whether the CD concentrations, the exposure time (incubation time), and their interactions affected the bioluminescence intensity of *A. fischeri*. CD concentration was considered a grouping factor, and the within-subject factor was the exposure time, which varied within the grouping factor. The Mauchley sphericity test was applied to confirm the criteria. Statistical analyses were performed at the *p* < 0.05 significance level. Tukey’s honestly significant difference test (Tukey HSD test) was used to compare the effects of the treatments. The significant effects are marked by an asterisk (*) in all figures (*p* < 0.05).

Pearson Product Moment Correlation Analysis was also performed by TIBCO Statistica™ 13.5 (TIBCO Software, Inc., Palo Alto, CA, USA) to examine the relationship between the measured endpoints and the cyclodextrin concentrations. The level of significance was *p* < 0.05. The correlation was considered strong when the correlation coefficient (r) was higher than 0.60 and very strong at r > 0.85.

Effective concentration (EC_20_, EC_50_) values causing 20 and 50% inhibition of bioluminescence intensity were determined using OriginPro 2018 software following the concentration-response analysis with Logistic function fitting (y = A2 + (A1 − A2)/(1 + (x/x0)^p^)). The determination of the concentration–response relationship is a method employed in the field of environmental toxicology, with the objective of objectively assessing the effects of different chemicals. The effective concentration endpoints (EC_20_ and EC_50_ values) of the tests are uniformly used for the quantification and comparison of the effects of chemicals and environmental matrices [23,41,42].

## 5. Conclusions

One possible approach to inhibit bacterial communication through quorum sensing is to prevent signal molecules from interacting with their receptors. This can be achieved by trapping the signal molecules, which will be unable to bind to the receptors and will go unnoticed by other microbes. Novel derivatives of cyclodextrin (CD) were synthesized to perform a dual function as traps for signal AHL molecules in the model organism *A. fischeri* and as ligands capable of mimicking the structure of these signal molecules via their alkyl chains, allowing them to bind to the AHL receptors to an appreciable extent. The autoinducer-dependent quorum-sensing mechanism in *A. fischeri* was found to be significantly suppressed by the application of cyclodextrins (CDs), which demonstrated pronounced quorum-quenching effects. The degree of efficacy was observed to be influenced by a range of parameters, including the dimensions of the interior cavity, structural composition, concentration of CDs, and the duration of exposure to the cells. The length of alkyl chains has been found to influence the aggregation behavior of molecular compounds. Specifically, when longer chains are present, micelle-like aggregates and large nanoparticles are formed. In this study, we examine the compounds with alkyl chains of C10–C16 length.

However, it is recommended that investigations be extended to the lower chain length region, where both solubility and aggregation may prove more advantageous. The study has yielded an important finding regarding the impact of QA groups on molecules. Specifically, it has been observed that though the presence of such groups enhances molecule solubility, it simultaneously results in a reduction in QS inhibition potential. The utilization of a specialized cyclodextrin trap designed to selectively complex the signal molecules produced by target microorganisms represents a promising strategy for interfering with their quorum-sensing (QS) processes. This approach holds the potential to enhance the efficacy of diverse biotechnologies, while simultaneously offering an alternative means of mitigating bacterial proliferation and infections. Moreover, our findings serve as a valuable point of departure for future research efforts targeted at different bacterial model systems.

## Figures and Tables

**Table 1:** RM ANOVA results of α-CD (**1**), QA-α-CD (**2**), and their alkylthio-substituted derivatives on the bioluminescence. Bold numbers indicate significant differences at *p* < 0.05, as determined by the Tukey HSD test of the Statistica™ 13.5 software.

Source of Variation	Df ^1^	MS ^2^	F ^3^	*p* ^4^
α-CD (**1**) treatment	4	7.94 × 10^7^	0.51	0.73
Time	**2**	**7.57 × 10^9^**	**570.07**	**0.00**
Time × α-CD (**1**) treatment	**8**	**8.12 × 10^7^**	**6.12**	**0.00**
6S-C12-α-CD (**3**) treatment	**4**	**7.75 × 10^9^**	**88.86**	**0.00**
Time	**2**	**1.54 × 10^9^**	**186.96**	**0.00**
Time × 6S-C12-α-CD (**3**) treatment	**8**	**1.43 × 10^8^**	**17.37**	**0.00**
QA-α-CD (**2**) treatment	4	1.02 × 10^8^	0.62	0.65
Time	**2**	**4.48 × 10^9^**	**322.40**	**0.00**
Time × QA-α-CD (**2**) treatment	**8**	**4.27 × 10^7^**	**3.07**	**0.00**
QA-6S-C10-α-CD (**4**) treatment	**4**	**2.60 × 10^9^**	**22.00**	**0.00**
Time	**2**	**2.87 × 10^9^**	**353.13**	**0.00**
Time × QA-6S-C10-α-CD (**4**) treatment	**8**	**5.78 × 10^7^**	**7.10**	**0.00**

^1^ Degree of freedom; ^2^ mean square; ^3^ F-ratio, ^4^ *p*-value.

**Table 2:** RM ANOVA results of β-CD (**5**), QA-β-CD (**6**), and their alkylthio-substituted derivatives on the bioluminescence. Bold numbers indicate significant differences at *p* < 0.05, as determined by the Tukey HSD test of the Statistica™ 13.5 software.

Source of Variation	Df ^1^	MS ^2^	F ^3^	*p* ^4^
β-CD (**5**) treatment	4	3.70 × 10^9^	2.59	0.05
Time	**2**	**8.94 × 10^9^**	**48.18**	**0.00**
Time × β-CD (**5**) treatment	8	9.67 × 10^7^	0.52	0.84
6S-C12-β-CD (**7**) treatment	**4**	**2.38 × 10^9^**	**22.35**	**0.00**
Time	2	5.74 × 10^6^	0.68	0.51
Time × 6S-C12-β-CD (**7**) treatment	**8**	**1.50 × 10^8^**	**17.60**	**0.00**
QA-β-CD (**6**) treatment	**4**	**7.68 × 10^9^**	**5.01**	**0.00**
Time	**2**	**6.25 × 10^9^**	**40.00**	**0.00**
Time × QA-β-CD (**6**) treatment	8	1.89 × 10^8^	1.21	0.31
QA-6S-C10-β-CD (**8**) treatment	**4**	**2.80 × 10^10^**	**33.18**	**0.00**
Time	**2**	**2.53 × 10^9^**	**15.48**	**0.00**
Time × QA-6S-C10-β-CD (**8**) treatment	8	2.59 × 10^8^	1.58	0.15
QA-6S-C16-β-CD (**9**) treatment	4	2.85 × 10^9^	2.44	0.07
Time	**2**	**5.32 × 10^9^**	**33.89**	**0.00**
Time × QA-6S-C16-β-CD (**9**) treatment	**8**	**6.49 × 10^8^**	**4.13**	**0.00**

^1^ Degree of freedom; ^2^ mean square; ^3^ F-ratio, ^4^ *p*-value.

**Table 3:** Effective concentration (EC) values of α- and β-CDs resulting in 20% and 50% inhibition of bioluminescence after 120 min exposure.

	Effective Concentrations [mM]—120 min	
	EC_20_	EC_50_
α-CD (**1**)	0.08	nd
6S-C12-α-CD (**3**)	0.06	0.16
QA-α-CD (**2**)	3.14	nd
QA-6S-C10-α-CD (**4**)	0.20	0.24
β-CD (**5**)	nd	nd
6S-C12-β-CD (**7**)	0.09	0.76
QA-β-CD (**6**)	nd	nd
QA-6S-C10-β-CD (**8**)	2.19	2.26
QA-6S-C16-β-CD (**9**)	0.17	nd

nd: not determined due to non-conventional concentration–response relationship.

**Table 4:** Chemical properties of the tested α-cyclodextrins.

α-Cyclodextrins (α-CDs)	A ^1^	No.	MW ^2^ [g/mol]	WS ^3^ [g/L]	DS ^4^
Native α-CD	α-CD	**1**	972	145	-
*N*,*N*,*N*-Trimethylammoniopropyl-α-CD *	QA-α-CD	**2**	1430	>500	2.5–4
6-monodeoxy-6-monododecanethiol-α-CD	6S-C12-α-CD	**3**	1188	>10	~1
*N*,*N*,*N*-Trimethylammoniopropyl-6-monodeoxy-6-monodecanethiol-α-CD	QA-6S-C10-α-CD	**4**	1160	~10	~1 (R-S) ~2 (QA)

^1^ Abbreviation; ^2^ molecular weight; ^3^ water solubility at 25 °C; ^4^ degree of substitution. * Commercial product of CycloLab Ltd.

**Table 5:** Chemical properties of the tested β-cyclodextrins.

β-Cyclodextrins (β-CDs)	A ^1^	No.	MW ^2^ [g/mol]	WS ^3^ [g/L]	DS ^4^
Native β-CD	β-CD	**5**	1135	18	-
*N*,*N*,*N*-Trimethylammoniopropyl-β-CD *	QA-β-CD	**6**	1665	>500	3–4
6-monodeoxy-6-monododecanethiol-β-CD	6S-C12-β-CD	**7**	1350	>10	~1
*N*,*N*,*N*-Trimethylammoniopropyl-6-monodeoxy-6-monodecanethiol-β-CD	QA-6S-C10-β-CD	**8**	1320	~10	~1 (R-S) ~2 (QA)
*N*,*N*,*N*-Trimethylammoniopropyl-6-monodeoxy-6-monohexadecanethiol-β-CD	QA-6S-C16-β-CD	**9**	1410	~10	~1 (R-S) ~2 (QA)

^1^ Abbreviation; ^2^ molecular weight; ^3^ water solubility at 25 °C; ^4^ degree of substitution. * Commercial product of CycloLab Ltd.

## Data Availability

The data that support the findings of this study are available on request from the corresponding author.

## Supplementary Materials

The supporting information can be downloaded at: https://www.mdpi.com/article/10.3390/ijms25137139/s1.
