# Supplementary material for: Long-Chain Alkylthio Cyclodextrin Derivatives for Modulation of Quorum-Sensing-Based Bioluminescence in Aliivibrio fischeri Model System
Source: Int J Mol Sci. 2024 Jun 28;25(13):7139. doi: 10.3390/ijms25137139 (PMC11241527; doi:10.3390/ijms25137139)
Supplement: Supplementary file 1 [file ijms-25-07139-s001.zip › ijms-2999306-supplementary.pdf]

SUPPLEMENTARY MATERIAL

Long-chain alkylthio cyclodextrin derivatives for modulation of quorum-sensing-based bioluminescence in *Aliivibrio fischeri* model system

Éva Fenyvesi, Zsófia Berkl, Laura Ligethy, Ildikó Fekete-Kertész, Márton Csizmazia, Milo Malanga, István Puskás, Levente Szőcs, Róbert Iványi, István Kese, Erzsébet Varga, Lajos Szente, Mónika Molnár

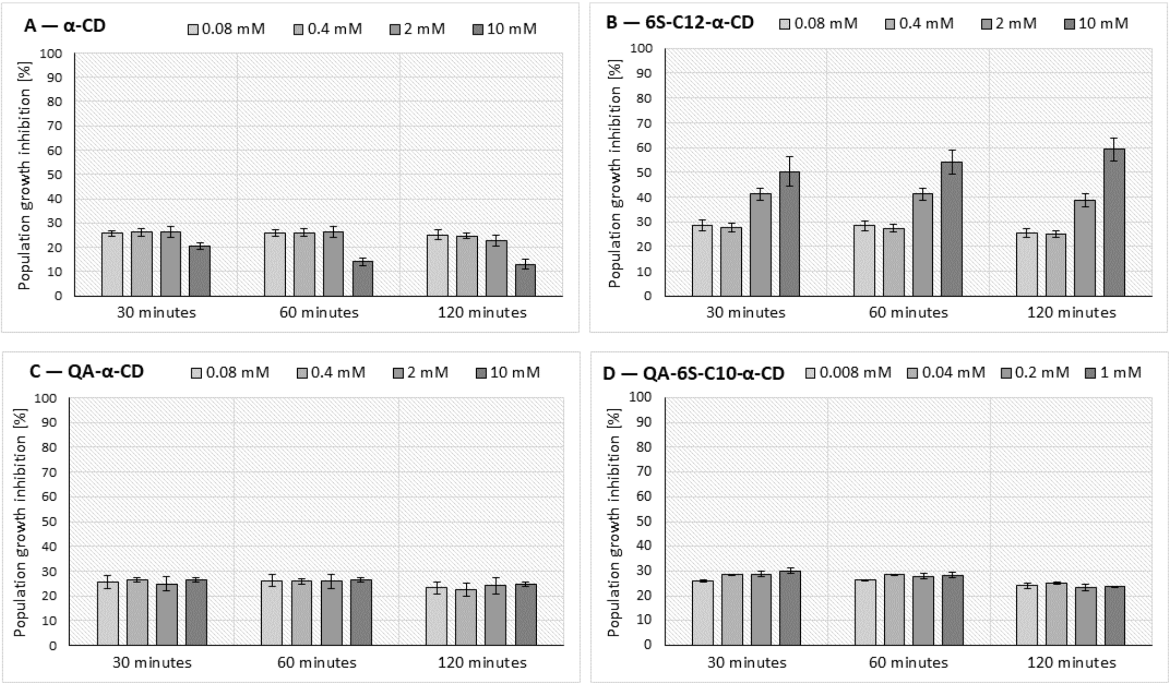

Figure S1. Effect of α-CD (A), 6S-C12-α-CD (B), QA-α-CD (C), and QA-6S-C10-α-CD (D) on the population growth of the *A. fischeri* after 30, 60, and 120 minutes of exposure. Data represent averages of four replicates.

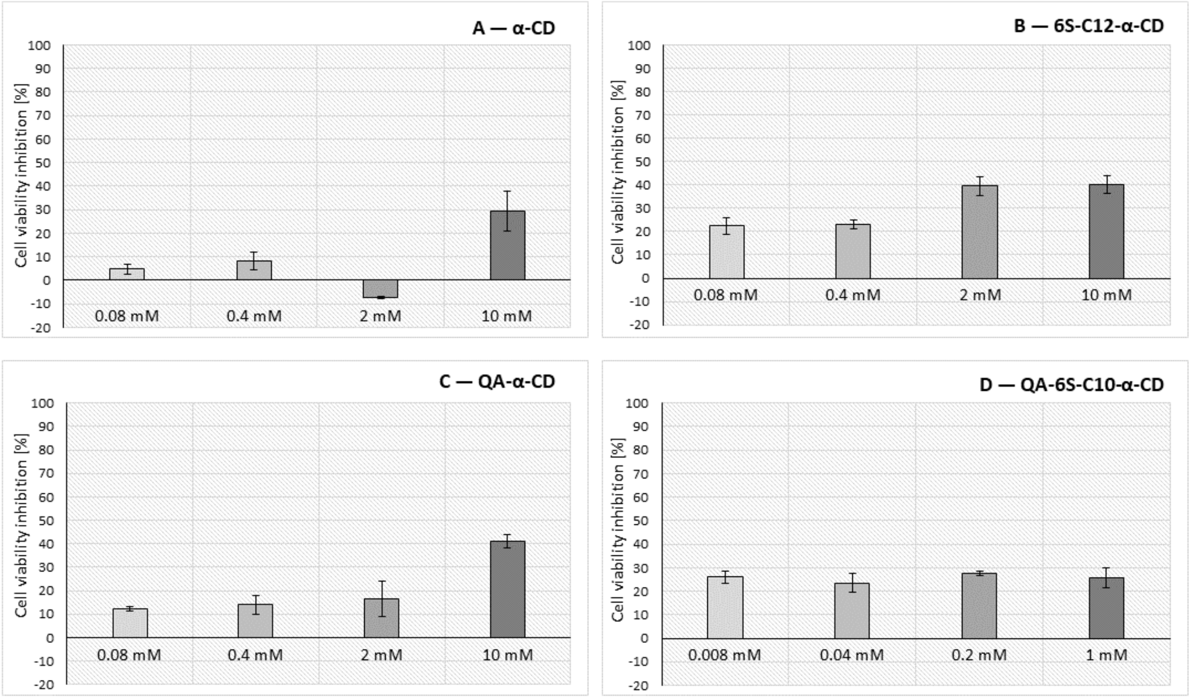

Figure S2. Effect of α-CD (A), 6S-C12-α-CD (B), QA-α-CD (C), and QA-6S-C10-α-CD (D) on the cell viability of the *A. fischeri* after 120 minutes of exposure. Data represent averages of four replicates.

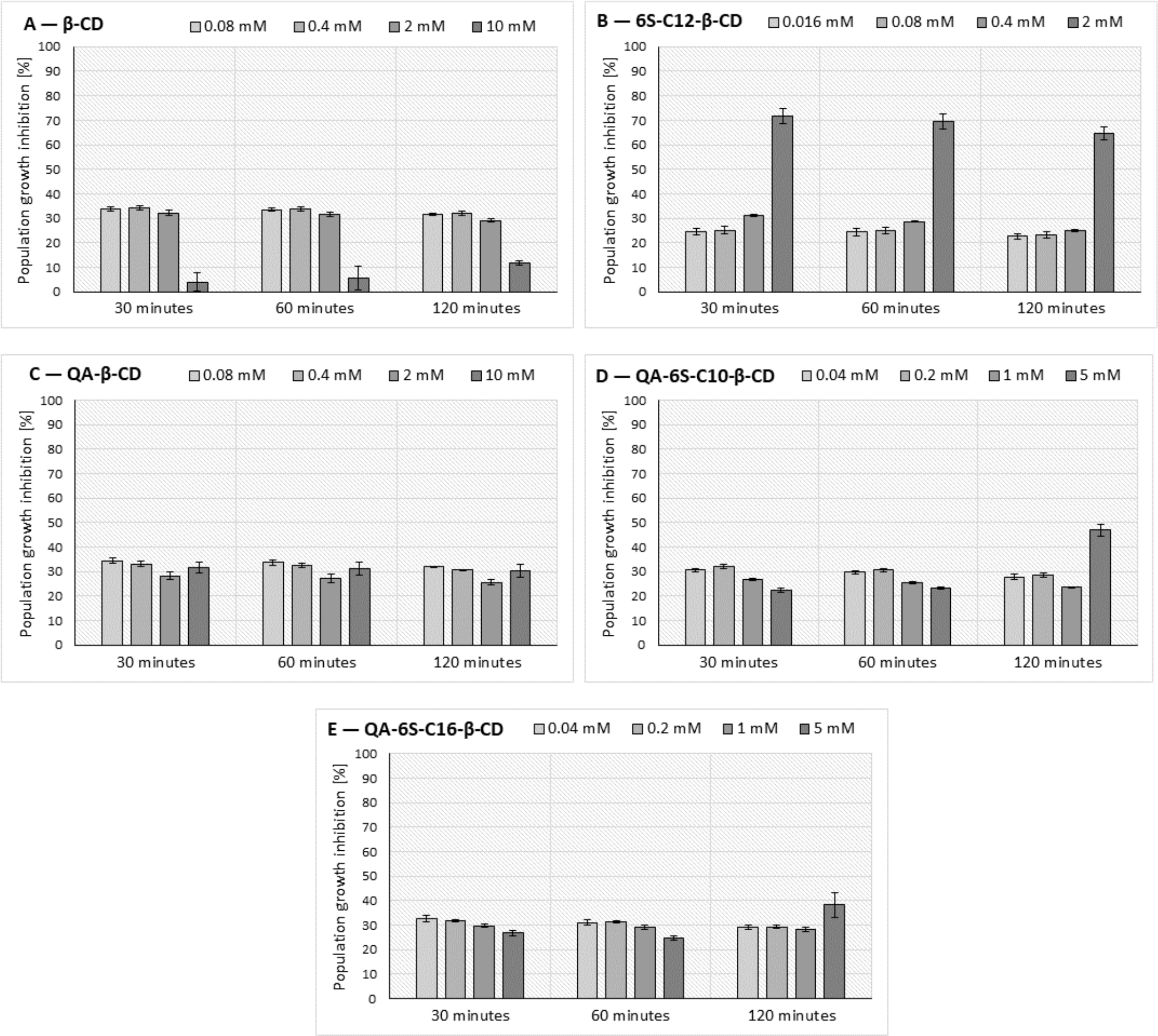

Figure S3. Effect of β-CD (A), 6S-C12-β-CD (B), QA-β-CD (C), QA-6S-C10-β-CD (D), and QA-6S-C16-β-CD (E) on the population growth of the *A. fischeri* after 30, 60, and 120 minutes of exposure. Data represent averages of four replicates.

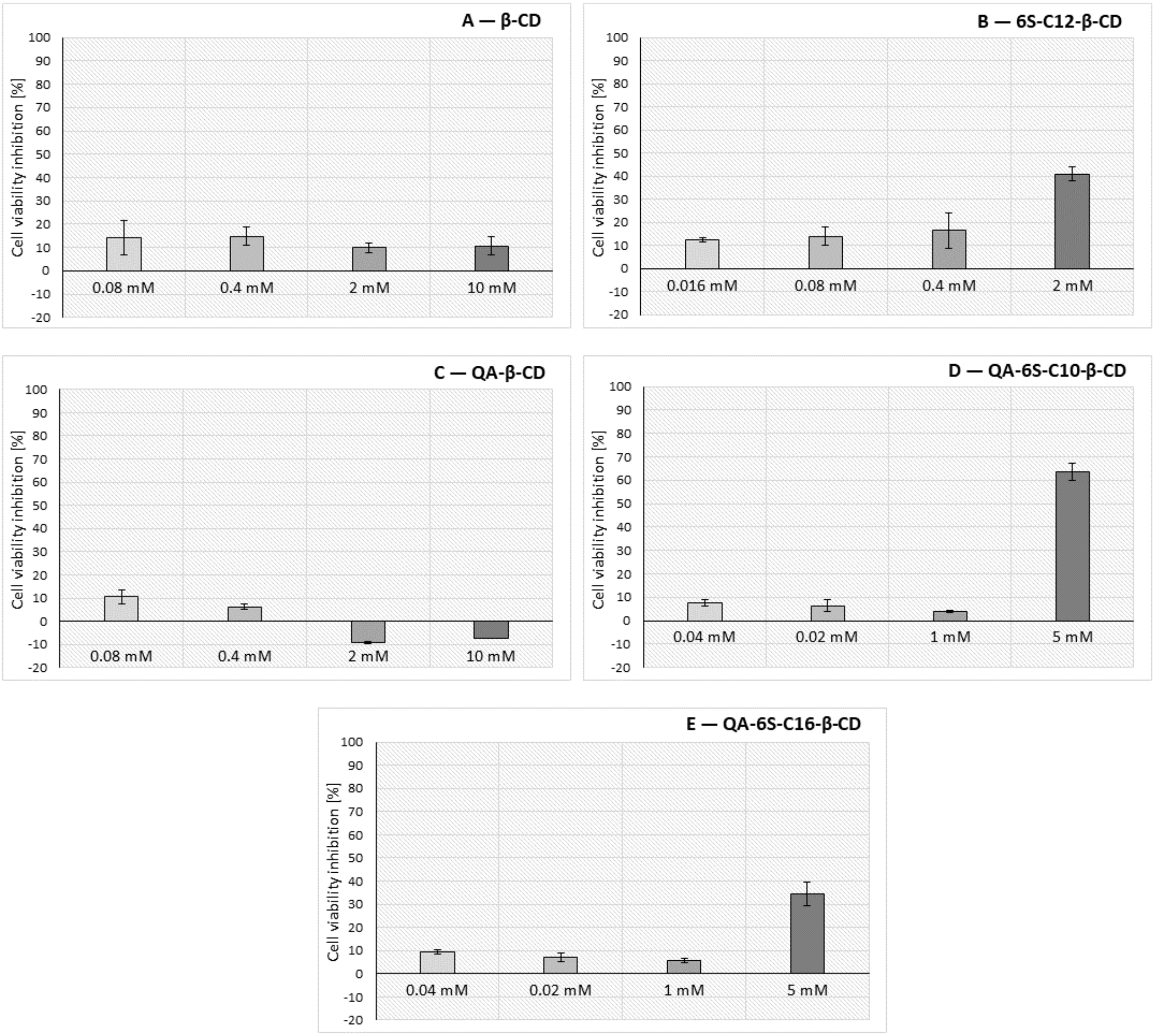

Figure S4. Effect of β-CD (A), 6S-C12-β-CD (B), QA-β-CD (C), QA-6S-C10-β-CD (D), and QA-6S-C16-β-CD (E) on the cell viability of the *A. fischeri* after 120 minutes of exposure. Data represent averages of four replicates.
